# Involving Adolescents in Decision‐Making in Pediatric Organ Transplantation: A Qualitative Study in Switzerland

**DOI:** 10.1111/petr.70146

**Published:** 2025-08-05

**Authors:** Alana Vallo‐Sacchettini, Cristina Zimmermann, Olivier Janjic, Michael Rost, Bernice Elger

**Affiliations:** ^1^ Institute for Biomedical Ethics University of Basel Basel Switzerland; ^2^ Department of Otolaryngology, Head and Neck Surgery Geneva University Hospital Geneva Switzerland; ^3^ Center for Legal Medicine University of Geneva Geneva Switzerland

**Keywords:** adolescents, bioethics, decision‐making, involvement, pediatrics, transplantation

## Abstract

**Background:**

In pediatrics, the triadic relationships between the adolescent, parents, and healthcare providers, as well as an adolescent's evolving developmental status, render decision‐making unique and challenging. The aim of this study was to explore adolescents' decision‐making preferences regarding pediatric organ transplantation.

**Methods:**

Semi‐structured one‐on‐one interviews were carried out with 17 patients aged 12–30 years (*M* = 20, *SD* = 5) who received organ transplants between the ages of 9 and 20 years (*M* = 13, *SD* = 3). The data were qualitatively analyzed via content analysis with a focus on an adolescent's involvement in decision‐making.

**Results:**

The participants considered various factors important for their appropriate involvement in decisions. These factors were grouped into three main areas: (1) a participant's role in decision‐making, (2) determinants of involvement in decision‐making, and (3) expression of the final decision. The most frequently described factors included the adolescent's age, concerns about what was happening to their body, the importance of social support systems, capacity to understand, and time given to adapt.

**Conclusions:**

According to Swiss law, being a minor does not deem adolescents incompetent; hence, adolescents can be legally competent in making medical decisions. Moreover, adolescents are the ones most affected by their illness and their respective treatment. It is therefore crucial to give a voice to them and to center their preferences in decision‐making. Our study shows that a range of distinct preferences concerning decision‐making and involvement exist among adolescents and, as such, can serve as points of leverage to improve the quality of care for this understudied patient population.

## Graphical abstract

This is the first interview study exploring the perspectives of transplanted adolescents on decision‐making regarding transplantation, and it shows that they want to be part of decision‐making at different levels of involvement.

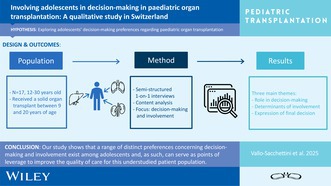

**Abbreviation**
HCPs: healthcare providers

## 1 Introduction

In pediatrics, the triadic relationship between the adolescent^1^, parents, and healthcare providers (HCPs), as well as an adolescent's evolving developmental status, renders decision‐making unique and particularly challenging. Adolescents are a group in a vulnerable position and are known to be “therapeutic orphans”, which means that they have historically been excluded from research and healthcare decision‐making [1, 2]. However, available evidence suggests that adolescents desire to be informed about their medical condition and treatment [3]. Furthermore, their ability to cope with upcoming medical procedures is enhanced when they are actively involved in the process [3, 4], such as being made aware of potential side effects [4]. Studies show that parents choose different degrees of involvement and often follow HCP advice even if it goes against their child's wishes [5]. For instance, in oncology, parents may follow the advice of HCPs to initiate chemotherapy even if their children wish to preserve their fertility, which would delay the start of treatment [4, 6, 7]. A hypothetical case study conducted by American researchers examining decision‐making for lung transplants in pediatric patients with cystic fibrosis shows that physicians will follow parents' choices regardless of the child's age or maturity [8].

Compared with a paternalistic approach, modern medicine focuses on patient autonomy. This, however, raises questions concerning the autonomy of adolescents [9, 10]. Ross and colleagues emphasized that adolescents have the right to make their own healthcare decisions in many U.S. states; however, most HCPs tend to side with parents in case of conflicts [11]. This is partially explained by the lack of education concerning the ability to appropriately involve adolescents, even if it has been proven to increase satisfaction with decision‐making and decrease conflicts between involved parties [12, 13]. Parents often attempt to protect their children and feel that this is an integral part of their role [6, 14]. Even if they let their children make their own decisions, parents' beliefs influence the decision‐making process. Hence, parents and HCPs must be aware of these relational aspects of autonomy [15, 16].

In Switzerland, the law (Swiss Civil Code, Art. 16) distinguishes patients with and without the capacity for judgment, irrespective of their age. Decision‐making capacity is defined by an adolescent's mental ability to understand the matter and to act in a reasonable way, which needs to be assessed in relation to a specific situation and for a specific question [17]. Respective legislation varies across countries and even within countries [18]. The European Council published a guide on children's participation in decisions about their health, emphasizing that the views of adolescents should be given due weight in accordance with their age and maturity [19]. According to Grootens‐Wiegers and colleagues, based on insights from numerous fields it can be concluded that adolescents at the age of 12 have the capacity to be decision‐making competent (i.e., understanding, appreciating, reasoning, communicating a choice); however, due to various factors diminishing adolescents' competence in some contexts, the authors argue that some adolescents may need support from facilitating environmental factors [20]. For urgent life‐limiting diseases, when adolescents are shaken by the diagnosis, time to assess their understanding might not be sufficient to establish whether they are fully capable of making decisions [21]. Yet, organ transplantation is often a question that arises after patients have lived with a disease for a few years, and with the progress of medicine, there can be acceptable alternatives to gain additional time [22, 23]. Thus, situations in which patients cannot be included should be rare.

As medicine evolves and the need for patient‐centered care grows, there is an increasing demand for research focusing on shared decision‐making. However, existing literature for the pediatric setting focuses more on shared decision‐making between HCPs and parents and hence the perspectives of children and adolescents are understudied [10, 24]. While many HCPs in the pediatric setting agree on the importance of involving adolescents in decisions about their healthcare, there is a gap in the literature concerning the views of children and adolescents themselves [21, 25]. Our study intends to fill this gap by giving voice to those with lived experience with pediatric organ transplantation. Transplant patients are chronic patients who tend to be familiar with the healthcare system and are faced with life‐and‐death decisions. The main focus was on participants' personal experiences and preferences regarding decision‐making as well as their attitudes toward involving adolescents in decision‐making. Our findings can serve as points of leverage to improve the quality of care and care experiences for this understudied population.

## 2 Materials and Methods

### 2.1 Study Design

We conducted a qualitative study with 17 semi‐structured interviews with participants from four Swiss university hospitals between June 2018 and February 2022. The study was approved by the competent cantonal research ethics committee (Nr. 2016–00444).

### 2.2 Participants

Participants were included if they had received a solid organ transplant before the age of 20 and were more than 9 years old at the time of the interview. This limit was chosen based on the experience of collaborating pediatric transplant specialists, the World Health Organization's definition of adolescence, and the cognitive development of adolescents. We deliberately chose a broad range to include the maximum number of developmental parameters related to decision‐making (e.g., age, diagnosis, prognosis, cause of disease, response to treatment) at the time of organ transplantation [26]. Another criterion was that the organ transplant occurred within the last 10 years for participants to be able to remember the process sufficiently. The interviews were carried out in French and German. Adolescents were excluded if they did not speak the same language as the interviewer did and if physicians did not consider them to be mentally and physically fit for participation. All patients gave their written consent to the person interviewing them. The parents of minor patients also gave their consent.

### 2.3 Recruitment

Participants were recruited with the help of head physicians. At the time of recruitment, 31 patients were eligible for the study. One of the collaborating physicians wanted to ask permission from their three patients before we contacted them. The remaining 28 were approached via telephone by the research team^2^. The background and aim of the study were explained to the participants. Ten of them refused to take part in the study without providing reasons. Four patients agreed initially but never found time for an interview. Since the research team had no contact with these patients prior to the study, and apart from approaching them to introduce the study, we cannot provide further details on why they have not participated. In total, 17 participants were interviewed.

### 2.4 Study Tool

A semi‐structured interview guide was developed by the research team on the basis of the available literature and expertise to capture experiences of and preferences regarding decision‐making and attitudes toward adolescents' involvement in decision‐making. The phrasing of the questions was adapted to the adolescents' capacity, as well as in light of their previous answers during the interviews. However, the main questions remained consistent throughout the study. A sample of these questions can be found in Table 1. The main areas of interest were their illness in general and how they learned about it, discussions about treatment options in relation to their illness, and their views on adolescents' medical decision‐making.

**TABLE 1:** Examples of questions.

**Time of diagnosis** Do you remember the first time you heard about your illness? Who told you about it? Did they tell you what was going to happen? Were there different treatment options? If yes, who explained them to you?
**Inclusion or exclusion of children** Please explain why you were included/excluded in the decision concerning your treatment? Who took the final decision concerning your treatment? Please explain. In which situation, and how, should children be included in the decision‐making? Please explain.

### 2.5 Data Collection

The interviews lasted between 10 and 60 min, were audio‐recorded, and were transcribed verbatim. Situations and names were anonymized. Transcripts were not returned to participants for comment. The interviewers were three team members, two women and one man, who were French and German speakers. Interviews were conducted at the time and place of the patient's choice, by video call for one patient and in person for 16 patients, which occurred in their home (*n* = 5), at the hospital (*n* = 6), or at another neutral place (*n* = 5) where they had follow‐up appointments. No differences in the data quality were noted. Short field notes were taken during the interview. The patients were only met once. Due to the adolescents' preference, seven patients had their parents present during the interview. Data collection continued until data saturation was reached.

### 2.6 Analysis

Content analysis was chosen to identify, analyze, and report relevant categories [27]. The analysis started with reading and rereading the transcripts. Line‐by‐line coding and analysis were performed via the software atlas.ti by two researchers. Transcripts were analyzed inductively. Identified categories were discussed, aiming to, first, establish a consistent understanding of the interview content (e.g., reflecting on researchers' possibly existing biases and positionality) and, second, specifying, revising, and agreeing on identified codes (including resolving conflicts through discussion). In the next step, approved codes were combined into three main themes and eight subthemes. Throughout analysis, codes and themes were continuously discussed with the entire team. All authors have experience with qualitative research; three worked as health care providers and two as researchers at the time of the study. This qualitative analysis enabled us to better understand how decision‐making regarding pediatric organ transplantation unfolds and how discussions were approached [28, 29].

## 3 Results

We interviewed 17 patients who received a solid organ transplant (liver, lung or kidney) between the ages of 9 and 20. The demographics of the participants and disease‐related information are presented in Table 2. The participants described their illness experience, their experiences with decision‐making, their perceived reasons for their degree of involvement, and their attitudes toward children's and adolescents' involvement in decision‐making. They also expressed their general opinion on the topic of decision‐making in pediatrics. Content analysis revealed three main themes and several subthemes related to decision‐making regarding organ transplantation: (1) the participants' role in decision‐making, (2) determinants of involvement in decision‐making, and (3) expression of the final decision. In the following paragraphs, we refer to participants with a random number, followed by the age they received the transplant and the type of organ in parentheses.

**TABLE 2:** Demographics and disease‐related information.

Organ	Sex (Male:Female)	Type of disease (I (congenital):II (acquired))	Type of donor Family:other	Age at transplantation (mean ± SD)	Age at interview (mean ± SD)
Liver	5:4	8:1^a^	0:9	12.5 ± 4.5	17 ± 6
Kidney	3:3	4:2^b^	2:4	14 ± 3	19 ± 5
Lung	2:0	2:0^c^	0:2	14.5 ± 2	23 ± 8

^a^ Cystic fibrosis, Wilson's disease, biliary atresia, Alagille syndrome, ascending cholangitis, Langerhans cell histiocytosis.

^b^ Nephronophthisis, Congenital anomaly, IgA Nephropathy.

^c^ Cystic fibrosis.

### 3.1 Participants' Role in Decision‐Making

#### 3.1.1 Presence During Decision‐Making

Most participants (*n* = 14/17) remembered that they were present during major conversations about their health or were aware of meetings that occurred without them at a time when they were not able to follow discussions because of the seriousness of their disease. When participants could not attend (*n* = 4/17), they found comfort in their parents' presence and did not feel excluded. They reported that they were involved in the discussions to varying degrees, most of the time based on their own preferences. Adolescents who described having more passive roles (*n* = 7/17) attributed their young age (*n* = 4/17) (less than 14 years old) as the reason for not being active, while others (*n* = 3/17) could not provide an explanation (Table 3).

**TABLE 3:** Quotes: Participants' role in decision‐making.

Subthemes	Participant's quotes
Presence during decision‐making	Patient 5 (13^a^, kidney): «When we're younger, we're much less likely to understand what's happening to us, and parents have to be there.» Patient 7 (13, kidney): « My parents had many discussions with the doctors, and then they came back to tell me the things in a different way, perhaps. Because doctors have their work to do, it's true that sometimes the words were a bit crude or cold. I'd say to myself: “I don't understand a thing”. »
Being informed	Patient 7 (13, kidney): «I think it all depends on the person because if the person says they want to know everything then I do not think it is acceptable to do it behind their back.» Patient 8 (15, liver): «I had a discussion with the surgeon. She explained very well, with drawings, how it was going to work […]. Everything was very well explained.» Patient 6 (18, kidney): « No, I think I'd like to know it all, as far as I'm concerned, it's better to know it all even if I regret it later. »

^a^ 13 represents age at transplantation.

#### 3.1.2 Being Informed

The participants' opinions on the content and amount of information received varied. They all agreed that they had the right to know everything if they asked for it (Table 3). However, not all (*n* = 5/17) expressed a desire to have complete information.

Several participants (*n* = 5/17) mentioned that they did not understand everything during technical conversations between HCPs, parents, and themselves. The participants who had more passive attitudes (*n* = 7/17) said that they were content with the fact that their parents were present to play the intermediary, to advise them, or to explain later the content of meetings using other terms. In contrast, those who wanted to be more actively involved (*n* = 10/17) expressed their wish to fully understand the discussion's content and were sometimes frustrated when this was not the case. The participants (*n* = 7/17) appreciated when physicians took the time to explain things in a manner that enabled them to understand what was going to happen (Table 3).

### 3.2 Determinants of Involvement in Decision‐Making

#### 3.2.1 Patient Age

The participants (*n* = 12/17) agreed that age is a key factor in determining the extent to which adolescents should be involved in their overall medical care (Table 4). Nevertheless, they believed that it is always connected with other factors, such as decision‐making capacity, maturity, or time lived with the disease. Moreover, the participants shared different points of view about the role of age. Some (*n* = 7/17) expressed the view that minor patients should never make medical decisions on their own. Others (*n* = 7/17) said that it is rather the capacity to comprehend than age per se that matters, and that this capacity does not come at the same age for everyone. When asked about what a suitable cut‐off could be, adolescents had very different points of view. Those involved (*n* = 10/17) in the decision‐making process estimated the age at which adolescents were capable of decision‐making to be younger than their own. Two participants set the age at 12, two others set it at 15, and one set it at 17. It was difficult to define an age for the younger patients who were less involved.

**TABLE 4:** Quotes: Determinants of involvement in decision‐making.

Subthemes	Participant's quotes
Patient age	Patient 9 (11, liver): «The age, I think. At 13–14‐15 years old, you understand what's going on, even if you do not want to, you understand it. » Patient 1 (15, Liver): « I don't think it necessarily has anything to do with age. I know there are plenty of people my age who are in good health, but if they become seriously ill, they don't necessarily have the experience. I've had a lot of treatments and I know how things work. »
Social support system	Patient 10 (16, lung): «Because doctors have their point of view, but as a patient you are happy to have a patient who talks to you about it. » Patient 10 (16, Lung): « I had a lot of problems with my mother because she suffered a lot. And she knew that I was in a very bad shape and that everything was really bad and there was almost nothing she could do. »
My body, my choice	Patient 5 (13, Kidney): «Children should always have a say. It is their life, their body, their illness. It belongs to them.» Patient 7 (13, kidney): «The doctors also said, “Listen, there's no point in arguing, we won't do it if she's not ready, because it is not going to go well.”» Patient 8 (15, Liver): « I would have felt that I was no longer in control of my body and that I was no longer in charge. And I'm glad the doctors came to me, because my parents tried to influence me by taking another treatment. »
Decision‐making capacity and comprehension	Patient 7 (13, kidney): Is it a good thing to always talk to the child (about the decisions to be made)? «Yes, but they need to be fully aware of the consequences of each choice, each option.» Patient 5 (13, kidney): «Yes, I think we can always do more. They explain with simple words, we manage to understand but I think that a child of 10, for example, we should perhaps explain with drawings or other.» Patient 6 (18, kidney): «When they first talked to me about my mother giving me a kidney, I refused. The doctors had a bit of trouble understanding why, understanding my choice. However, then I tried to explain why, and they agreed.»
Time given to adapt to the diagnosis	Patient 1 (15, liver): «I have been treated since I was a child, I have had a lot of treatments and I know how things work. Someone who's never had any treatment will not necessarily have the experience to make the right decision.» Patient 17 (15, Liver): « I think people who are born with a disease are prepared for it and they know it. »

#### 3.2.2 Social Support System

A social support system often provided by family members was deemed crucial for adolescents (*n* = 6/17). All participants talked about the roles and support of their parents and family members – “My grandparents, my aunts and uncles came to visit. I'm lucky to have a close‐knit family, so I've had a lot of support from my family.” (Patient 7, 13, kidney). At the same time, they talked (*n* = 5/17) about the intrafamilial difficulties (e.g., siblings left alone at home) that may arise from their disease. They (*n* = 6/17) also emphasized the importance of feeling close to their families while in hospital (Table 4). Patient 10 (16, lung) also had the support of a fellow patient through a hospital “buddy system”, which allowed them to share experiences, ultimately helping the adolescent make decisions (Table 4).

#### 3.2.3 My Body, My Choice

Regarding reasons for their inclusion in decision‐making, most participants (*n* = 8/17) stated what was emphasized by patient 4 (16, kidney): “because it was my health that was at the centre of it all, it concerned me.” Even if sometimes, they could not make their own decisions, adolescents always wanted to be informed about the options and wanted their decisions and wishes to be taken into consideration, regardless of their age, as it was about their body and life.

Some (*n* = 3/17) even expressed that forcing someone to undergo an unwanted medical procedure could have important repercussions and compromise the success of the procedure. One participant reported that the physician in charge also agreed with this line of reasoning (Table 4). Some participants (*n* = 2/17) also stated that forcing them into a medical procedure that they would not have wanted would have caused them to feel that they were no longer in charge of their own body. This happened to two patients, who remembered it as traumatic. For example, one patient woke up naked with a urinary catheter, but no one explained what was going to happen.

#### 3.2.4 Decision‐Making Capacity and Comprehension

The participants were convinced that the capacity to understand the details of a decision is central to the ability to make a proper decision (Table 4). However, they had different ideas on what influenced this capacity. For some (*n* = 4/17), it was better to let the doctor, who had medical expertise, decide on the best option. For others (*n* = 12/17), age had a great deal to play in the ability to understand or the experience that comes with dealing with an illness daily. Patient 5 (13, kidney) also stated that HCPs should explain details in different ways for the adolescent to be able to understand (Table 4).

A patient explained that providing argumentation can be a form of assessment regarding whether an adolescent has understood the pros and cons of the transplantation and is therefore able to decide. According to this participant, patients should be taken seriously if they are able to explain their arguments. If the argumentation is valid, there is no reason not to listen, regardless of age (Table 4).

#### 3.2.5 Time Given to Adapt to the Diagnosis

Some participants (*n* = 6/17) reported that their lived experience of the illness helps in the decision‐making process. At the time of diagnosis, their life changed; there was much information to process, and it was very difficult, if not impossible, to make decisions at that time. Once they learned to live with their disease, became accustomed to medical appointments, and familiarized themselves with some of the vocabulary related to their medical condition, their ability to make their own decisions increased (Table 4).

### 3.3 Expression of the Final Decision

#### 3.3.1 Sharing the Decision

All the participants (*n* = 17/17) expressed the wish to make the decision together with their family and the involved HCP. They had a strong need to feel their family's support during this process. They agreed that their health choices are complicated and that it is reassuring that family members are included and agree with the decision (Table 5).

**TABLE 5:** Quotes: Expression of the final decision.

Subthemes	Participant's quotes
Sharing the decision	Patient 6 (18, kidney): «I think that before talking to the doctors, you have to talk with your family, you have to make the decision together.» Patient 13 (14, Liver): « Everyone was of the same opinion and came to the same conclusion: Yes, that's good. »
The adolescent making the final decision	Patient 17 (15, liver): «So I think it goes without saying that we as a person should be allowed to make our own decision, regardless of whether the parents say yes or no or the doctors say yes or no. I actually think that the child should be allowed to decide, because we are also cut open afterwards and something is taken from us.» Patient 5 (13, Kidney): « If I had to do it all over again, at my age now, I'd like it to be me who really says the answer, yes or no. Even if I think it's going to be yes, I'd like it to come out of my mouth more than that of my parents or doctor. »

#### 3.3.2 The Adolescent Making the Final Decision

Furthermore, for the adolescents (*n* = 8/17) who wanted to hold an active role in decision‐making, there was a need to be listened to, to be given the chance to express themselves, and to actually make the final decision (Table 5).

## 4 Discussion

To our knowledge, this is the first empirical study worldwide to explore the perspectives of transplanted minors and the first publication on adolescents' views in Europe. Christian and colleagues published a case report concerning one adolescent patient with cystic fibrosis and his journey through decision‐making leading to transplantation in the USA. Notably, this patient actively participated in determining the appropriate timing for the transplant and was involved in the decision‐making process [30]. Our study confirms how important it is for adolescents to be involved and able to make decisions about their health care.

Our participants emphasized the importance of making major decisions together with their parents and HCPs, similar to minors outside the field of transplantation [31]. They described their degree of involvement as varying depending on their age, knowledge, experience, personality, and most importantly, on their preferences, which has also been reported in another Swiss study in pediatric oncology [3]. HCPs should consider our finding that some adolescents want to play a passive role, whereas others want to be able to hold an active role, which is their right [32, 33]. To better exercise their right to be involved and able to make decisions, patients need to understand the options available to them [34]. This is in line with current concepts of decision‐making capacity, defined as the capacity to provide informed consent after assimilating information and understanding and balancing it [20, 35, 36]. It is important for HCPs and parents to be aware that their beliefs influence the adolescent's decision and that it is imperative to provide adolescents with explanations and communication skills to make decisions [15]. As stated by one of the interviewees, the capacity to argue one's point of view can be a way to assess patients' decisional capacity. However, this is a challenging task, especially for young adolescents. Yet, being able to explain in their own words would also provide HCPs with valuable insight into what has been understood. It is also known that patients who are informed early about their health cope better with their diagnosis [37]. This allows them to adjust to their state of health and this new life.

HCPs should proactively inquire about adolescents' preferences and take time to explain details in a developmentally appropriate manner. The ability of adolescents to understand and appreciate information, and argue their point of view, evolves over time, depending on factors such as the time since diagnosis, age, neurological development, and illness experience [20]. Hence, physicians should continuously reassess adolescents' capacity and wishes regarding involvement in decision‐making [4, 38]. Several studies note that teenagers who have been treated in the medical system for several years and have always been cared for might assume that their role is passive [3, 4, 39]. This means that reassessing their preferred level of involvement is necessary not only to understand their changing preferences but also to enhance their autonomy. Most participants also stressed their wish to have the chance to express their opinion. It is also an opportunity for physicians to better understand the adolescent's understanding and preferences regarding their healthcare [40, 41].

Concerning the relationship between age and a patient's preferred degree of involvement, our findings show that there is no rule of thumb that would enable HCPs to exactly know how to proceed. It is generally agreed that by “the age of 14 to 15 years, most young people possess the capacity to make treatment decisions” [30], but our study shows that not all adolescents agree with this when they face a life‐changing decision. Indeed, there is great variability in the age at which patients want to be able to make the decision, with some adolescents wanting to do so before the age of 14. We found that the included adolescents agreed that the illness experience and the capacity to comprehend it are crucial determinants of a patient's decisional capacity. Both depend on many factors in addition to age and thus must be assessed carefully for each individual. Relatedly, studies have shown that HCPs need more training to be able to better support patients and their families in their decision‐making [42, 43].

Future research should investigate strategies to assist healthcare professionals in involving patients in a manner tailored to their individual capacity and preferences, while also effectively communicating this approach to parents [7, 44]. Furthermore, there is a need for age‐adapted communication tools. HCPs should take seriously the suggestions of our participants that talking is one way of communicating; but, for example, drawing, as suggested by two of our participants, can be another more suitable way for adolescents. We could also imagine other tools such as videos, stories, or podcasts. The need for age‐adapted tools to assess decision‐making capacity is even more important in Switzerland, where capacity (not age) is the basis on which professionals rely to know whether a patient is legally competent.

### 4.1 Limitations

First, as the mean time between transplantations and interviews was five years, some recall bias probably exists. In addition, as the period between transplantations and interviews was long, the participants gained in maturity, which could have biased their recall toward a more positive view of the decision‐making process. However, as transplantation is a critical life event, recall bias is assumed to be minor. Second, since patients were selected by their physician to participate in our study, we cannot exclude possible selection bias, especially since one of the physicians talked to his patients first. Indeed, refusal to participate in our study for “personal matters” could be caused by a complicated relationship between patients and HCPs, dissatisfaction with decision‐making, or parents' opposition to participate rather than adolescents' choices. However, the exclusion of adolescents with lower satisfaction could mean dissatisfaction due to both too little or too much involvement in decision‐making (or other reasons). The motivation to participate in the study could also be influenced either by satisfaction or dissatisfaction with care and decision‐making; thus, we have no reason to believe that the selection was biased toward patients who were more positive toward involvement in decision‐making. Third, our sample is composed of different organ transplants (liver: *n* = 9, kidney: *n* = 6, lung: *n* = 2), and adolescents with a liver transplant were younger. While we did not identify differences in answers between groups, possible differences could be addressed with future research, particularly for age.

## 5 Conclusion

Although involving adolescents in decision‐making is an ethico‐legal imperative and has been shown to increase satisfaction, it is not yet common practice in all hospitals and for all settings [5, 34]. Our study explores the views of transplanted adolescents who went through the decision‐making process. This shows that they want to be part of decision‐making and choose their level of involvement. Practice should involve patients unless they wish otherwise. Their wish to be involved is often driven by many factors, such as decision‐making capacity, maturity, or time lived with the disease. Their social support system also plays a critical role. Importantly, patients are those most directly affected by their disease and related treatment. Therefore, it is essential that they are provided with opportunities to articulate their preferences and make informed choices regarding their care. Lastly, to effectively enhance adolescents' autonomy, areas for future research are: differences regarding decision‐making preferences across various groups of adolescents (e.g., age, transplanted organ, cultural factors), communication tools facilitating decision‐making surrounding pediatric organ transplantation, and a longitudinal assessment of adolescents' perspectives before and after such decisions.

## Author Contributions

A.V.‐S.: formal analysis, investigation, data curation, writing – original draft, visualization. C.Z.: formal analysis, investigation, data curation. O.J.: conceptualization, methodology. M.R.: writing – review and editing, supervision. B.E.: Conceptualization, methodology, supervision, project administration, funding acquisition.

## Ethics Statement

The study was approved by the competent cantonal research ethics committees (Nr. 2016–00444).

## Consent

Informed consent was obtained from all individual participants included in the study.

## Conflicts of Interest

The authors declare no conflicts of interest.

## Data Availability

The data that support the findings of this study are available on request from the corresponding author. The data are not publicly available due to privacy or ethical restrictions.

## Endnotes

^1^
Throughout this article, the terms “adolescent” and “adolescents” are used to refer to the ages between 9 and 20 as this represents the range of our sample.

^2^
Underage patients were approached through their parents.
